# Assessment of Patient Satisfaction With Primary Septorhinoplasty Using the Rhinoplasty Outcome Evaluation Questionnaire

**DOI:** 10.7759/cureus.11777

**Published:** 2020-11-29

**Authors:** Ergin Bilgin, Mehmet Ali Say, Deniz Baklacı

**Affiliations:** 1 Otolaryngology, Bülent Ecevit University Hospital, Zonguldak, TUR; 2 Otolaryngology, Ataturk State Hospital, Zonguldak, TUR

**Keywords:** septorhinoplasty, roe questionnaire, patient satisfaction

## Abstract

Objective: To evaluate patient satisfaction according to demographic characteristics using the Rhinoplasty Outcome Evaluation (ROE) questionnaire.

Methods: In this retrospective observational study, a total of 60 patients that underwent septorhinoplasty were evaluated. The ROE questionnaire was administered to evaluate patient satisfaction after septorhinoplasty.

Results: There were 24 (40%) males and 36 (60%) females in the study. The mean age of the patients was 32.5 years. In the whole sample, the mean postoperative ROE score was 87.9. It was observed that the mean postoperative ROE score of the patients aged 30 and below was lower compared to the >30 age group (p < 0.001). Furthermore, the mean postoperative ROE score was significantly higher in male patients than in females (p = 0.019).

Conclusion: We conclude that the ROE questionnaire is a simple and useful tool for evaluating septorhinoplasty outcomes. Demographic characteristics such as male gender and age > 30 are factors that positively affect the satisfaction of patients with septorhinoplasty.

## Introduction

The nose is very important due to its central position in the face. Nose shape is often one of the factors affecting personality development and body image. Patients usually have complaints pertaining to both aesthetic and functional aspects of the nose; therefore, septorhinoplasty is one of the most common surgical procedures performed for both functional and aesthetic purposes [1]. The demand for septorhinoplasty has significantly increased over the past two decades due to increased personal interest, media awareness, and advances in surgical techniques. This trend is most common in the younger age group for both men and women [2].

The quality of surgery, the surgeon’s level of expertise, and most importantly the patient’s level of expectation are important factors in patient satisfaction with septorhinoplasty. Patient satisfaction varies according to gender, age, education level, culture, ethnic origin, and last but not least patient expectation [3]. Patient selection in septorhinoplasty is very important since a significant percentage of patients may not be satisfied despite a good surgical outcome [4].

Traditionally, the surgical success of a septorhinoplasty procedure is mainly evaluated using anthropometric measurements and anatomical markers of facial measurements [5-7]. Although these measurements allow an accurate assessment of surgical results, they often overlook patient satisfaction, which is one of the most important determinants of the success or failure of this procedure. The Rhinoplasty Outcome Evaluation (ROE) questionnaire is a fast and easy-to-apply tool for the standard and reliable assessment of the quality of life after rhinoplasty. It measures qualitative aspects, such as social, emotional, and psychological variables [8].

This study aimed to determine the satisfaction level of patients that underwent septorhinoplasty in a tertiary center, using the ROE questionnaire preoperatively and postoperatively, and to identify the factors affecting patient satisfaction.

## Materials and methods

After obtaining the ethics committee approval (2019-204-18/12), a prospective observational study was conducted with patients that underwent primary septorhinoplasty for various reasons in our center between December 2019 and February 2020. Written informed consent was obtained from all patients before the operation. Patients aged 16-40 years who underwent septorhinoplasty with the open technique were included. The exclusion criteria were inability to understand the questionnaire items, age under 16 years, a history of revision surgery or closed rhinoplasty, and concurrent functional endoscopic sinus surgery or other nasal airway procedure. The demographic characteristics of all patients included in the study were recorded. The validated Turkish version of the ROE questionnaire consisting of six questions was used (Table 1). This tool contains five questions about nose shape and one question about nasal breathing. Each ROE question is answered on a scale of 0 to 4, with 0 indicating the most negative and 4 indicating the most positive response. The patients’ responses to each question were summed up, and a score varying between 0 (minimum satisfaction) and 100 (maximum satisfaction) was obtained by dividing the total by 24 and multiplying it by 100.

**Table 1:** ROE questionnaire ROE, Rhinoplasty Outcome Evaluation.

ROE Questionnaire				
1. How well do you like the appearance of your nose?				
Not at all (0)	Somewhat (1)	Moderately (2)	Very much (3)	Completely (4)
2. How much can you breathe through your nose?				
Not at all (0)	Somewhat (1)	Moderately (2)	Very much (3)	Completely (4)
3. How much do you think your friends and close ones like your nose?				
Not at all (0)	Somewhat (1)	Moderately (2)	Very much (3)	Completely (4)
4. Do you think your current nasal appearance limits your social or professional activities?				
Always (0)	Usually (1)	Sometimes (2)	Rarely (3)	Never (4)
5. How confident are you that your nasal appearance is the best it can be?				
Not at all (0)	Somewhat (1)	Moderately (2)	Very much (3)	Completely (4)
6. Would you like to surgically alter the appearance or function of your nose?				
Definitely (0)	Most likely (1)	Possibly (2)	Probably not (3)	No (4)

The ROE questionnaire was administered to all patients by the same researcher preoperatively and at the postoperative sixth-month follow-up. The data of all patients were compiled, and the results were compared in terms of gender (female, male) and age (≤30, >30) groups.

Statistical analysis

Mean and standard deviation values were used as descriptive statistics for continuous data, and the Shapiro-Wilk test was utilized to examine the conformance of continuous data to normal distribution. The dependent samples t-test was used to compare the ROE scores before and after surgery. The independent samples t-test was conducted to compare the preoperative and postoperative ROE scores according to gender and age groups. IBM SPSS Statistics v. 20 (IBM Corp., Armonk, New York) was used for statistical analyses, and p < 0.05 was accepted as the statistical significance limit.

## Results

Sixty of the 78 patients that were initially reviewed completed the questionnaires and were included in the study. Sixty percent (n = 36) of the patients were females, and 40% (n = 24) were males. The mean age of the whole sample was 32.5 ± 6.4 years. The mean age was 32.5 ± 9.2 years for women and 32.9 ± 5.3 years for men. Twenty seven (45%) of the patients were in the >30 years group and 33 (55%) in the ≤30 years group. In our study population, the reasons of septorhinoplasty were esthetic in 10% (n = 6), functional in 25% (n = 15), and combined in 65% (n = 39).

The mean preoperative and postoperative sixth-month ROE scores of the patients were 33.75 ± 6.45 and 87.9 ± 6.85, respectively. A statistically significant improvement was observed in the preoperative and postoperative mean ROE scores in the whole sample (p < 0.001). While there was no statistically significant difference in the preoperative mean ROE scores between the female and male patients (p = 0.943), the postoperative mean ROE score was statistically significantly higher in male patients than in females (p = 0.019) (Table 2).

**Table 2:** Comparison of preoperative and postoperative ROE scores between male and female patients ROE, Rhinoplasty Outcome Evaluation; SD, standard deviation.

ROE score (mean ± SD)	Gender		
	Female	Male	p Values
Preoperative	33.7 ± 6.58	33.6 ± 5.30	0.943
Postoperative	83.52 ± 6.52	89.54 ± 6.69	0.019

There was no statistically significant difference between the age groups in terms of the mean preoperative ROE scores (p = 0.153), whereas the mean postoperative ROE score was determined to be statistically significantly higher among the patients aged >30 years compared to the ≤30 years group (p < 0.001) (Table 3).

**Table 3:** Comparison of preoperative and postoperative ROE scores between age groups ROE, Rhinoplasty Outcome Evaluation; SD, standard deviation.

ROE score (mean ± SD)	Age group		
	≤30	>30	p Values
Preoperative	34.37 ± 6.67	32.63 ± 6.18	0.153
Postoperative	79.16 ± 6.37	91.25 ± 6.27	<0.001

## Discussion

Septorhinoplasty is a challenging procedure considering that the purpose of this surgery is not only to restore the function and original appearance of the nose but also to improve the quality of life. Over the last few decades, the trend has rapidly changed from more invasive procedures to less invasive procedures to achieve these aims. Although the technical aspects of septorhinoplasty are important, the main factor determining the success of the procedure is patient satisfaction [9]. A recent study suggested that meeting esthetic rather than functional expectations was more important to satisfy patients [10].

Measuring patient satisfaction is a difficult task since there are no real standards. Compared to other cosmetic procedures, patients undergoing rhinoplasty are usually less satisfied with their postoperative appearance [11]. Patient-reported outcome measures (PROM), which evaluate the quality of the treatment offered to patients from their perspective, have become increasingly popular in documenting the effectiveness of esthetic interventions using quantitative methods [12]. ROE is one of the validated PROM tools that have been reported to be useful and widely accepted for the assessment of patient satisfaction with rhinoplasty outcomes [13].

In this study, we chose to use the Turkish version of the ROE questionnaire as an easy-to-apply, short and validated tool. This questionnaire evaluates respiratory function, quality of life, and cosmetic results. The surgeon and the patient are not always equally satisfied with outcomes as expectations and opinions differ. Therefore, it is very important to understand the expectations of patients before surgery to ensure their satisfaction. Patient satisfaction may be affected by gender, age, social background, education level, and psychological status [14]. In a recent study by Esteves et al., the satisfaction and quality of life of patients were reported to be significantly improved after rhinoplasty. In our study, the mean ROE score was 33.75 ± 6.45 preoperatively and 87.9 ± 6.85 at the postoperative sixth month, and the mean postoperative increase in the ROE score was 54.1 ± 1.2 points. Gender, age, type of surgical approach, and additional nasal procedures were found to have no effect on postoperative satisfaction scores. In addition, patients with low literacy levels were found to be more satisfied with the procedure [8].

Age can be an important factor determining the patient satisfaction score. Balıkçı et al. and Litner et al. found lower satisfaction scores in younger patients and interpreted that this group had higher expectations and thus had difficulty accepting changes in their own image [15,16]. Arima et al. reported that satisfaction scores in patients under 30 years were lower than those aged over 30 years [17]. In our study, the postoperative ROE scores were significantly higher in the patients over 30 years of age compared to those aged 30 years and younger.

In our study, a higher surgery satisfaction score was found in men than in women (89.54 and 83.52, respectively). In contrast, previous studies show that the overall satisfaction rate after rhinoplasty is higher in women than in men [18-20]. Cingi et al. reported an improvement in the postoperative ROE scores of both genders but noted greater differences between the preoperative and postoperative ROE scores among men compared to women [21]. On the other hand, Sözen et al. found no significant relationship between the ROE score and gender in rhinoplasty patients [22].

Our study presented preliminary data on patient satisfaction after septorhinoplasty based on a single assessment tool. Further studies with larger sample sizes and more specific quality of life tools are needed to further confirm the benefits of septorhinoplasty. Another limitation of our study is that septorhinoplasty was performed by different surgeons with different specialization levels, and the patients’ biases on this issue may have affected the data obtained in the preoperative and postoperative periods. The difference between senior and junior residents can also have an impact on results since the level of expertise increases over time, especially over the course of training.

## Conclusions

Achieving 100% patient satisfaction is unrealistic, but awareness of new surgical techniques and patient requirements has developed over the past decade. PROMs are useful tools in evaluating the benefit of surgery from the patient’s perspective. In this study, we showed that demographic variables such as gender and age were potential determinants of the degree of patient satisfaction. We conclude that the ROE questionnaire is a simple and useful tool for evaluating septorhinoplasty outcomes.
